# Reconstruction of Large Full-Thickness Abdominal Wall Defects Using a Free Functional Latissimus Dorsi Muscle

**DOI:** 10.3389/fsurg.2022.853639

**Published:** 2022-03-17

**Authors:** Marijana Ninkovic, Marina Ninkovic, Dietmar Öfner, Milomir Ninkovic

**Affiliations:** ^1^Department of Visceral, Transplant and Thoracic Surgery, Center of Operative Medicine, Innsbruck Medical University, Innsbruck, Austria; ^2^Department of Plastic, Reconstructive, Hand and Burn Surgery, München Klinik Bogenhausen, Munich, Germany

**Keywords:** abdominal wall defect, functional reconstruction, hernia abdominalis, abdominal wall weakness, free functional latissimus dorsi flap

## Abstract

**Introduction:**

The large full-thickness abdominal wall defect has to be treated by considering anatomical and functional requirements. The abdominal wall must regain total physiological function, which means that the full thickness abdominal wall defect must be reconstructed anatomically, not only according to the anatomical requirements but also maintaining the functional dynamic voluntary movement. Defects in the abdominal wall alter respiratory mechanics and can impair the diaphragm function. Additionally, muscles of the anterolateral abdominal wall increase the stability of the lumbar region of the vertebral column by tensing the thoracolumbar fascia and by increasing intraabdominal pressure.

**Materials and Methods:**

The timing and method of reconstruction must be chosen depending upon the etiology of the defect. Severe traumatic injuries, abdominal wall infections, necrotizing soft tissue loss, or sepsis needs to undergo staged reconstruction following adequate debridement to control the infectious process, establish the zone of injury, and for proper treatment of intraabdominal pathology, thereby achieving temporary primary closure using split-thickness skin grafting to the viscera. At the time of definitive reconstruction, deep skin graft dermabrasion give us a facial-like layer with adequate strength to stabilize the static abdominal wall. This dermal layer is supported by free functional (innervated) latissimus dorsi muscle (fLDM), giving full anatomical coverage and functional stability. After oncologic resections full-thickness abdominal wall reconstruction was performed immediately with a combination of fLDM flaps and meshes.

**Results:**

A total of 14 patients underwent abdominal wall reconstruction using the fLDM flap. Staged reconstruction was applied in 8 cases. In the remaining six cases, two had no mesh support, three had synthetic mesh, and one had a fascial graft, which were covered with fLDM flap. There were no free flaps failure. One flap revision due to venous anastomosis thrombosis was performed. Donor site seromas occurred in 5 cases and were treated with punction and direct doxycycline injection. Electromyographic testing postoperatively confirmed reinnervation of transplanted LDM.

**Conclusion:**

Using fLDM as a definitive solution, we are not only able to repair soft tissue defects, but also reconstruct voluntary contractility and dynamic natural functional abdominal wall. Transplanted LDM offers enough contractile capacity and strength to replace the function of the missing abdominal wall muscles.

## Introduction

The large full-thickness abdominal wall defect must be treated by considering some decisive problems. The abdominal wall must regain total physiological function, which means that the full thickness abdominal wall defect must be reconstructed anatomically, maintaining the dynamic voluntary movement. The shrinking of the transferred tissue during the healing process might lead to respiratory restrictions and should therefore be avoided. According to anatomical function and esthetic requirements, abdominal wall defects continue to be a challenge for the reconstructive surgeon. Abdominal wall deficiency can be classified into two groups. The hernia, an abdominal wall weakness limited to the fascia but with adequate soft-tissue coverage, and the full-thickness abdominal wall defect that requires soft-tissue coverage. Both require repair by functional replacement of the missing tissue. In the cases of abdominal wall weakness, full stability can be achieved by using synthetic meshes (1–3) or autologous grafts (4, 5). The goals of reconstruction of the full-thickness abdominal wall include reestablishment of the functional integrity of the abdominal wall with adequate soft-tissue coverage (6). After Wangensteen (7, 8) reported the use of vascularized fascia lata to reconstruct the abdominal wall, numerous flaps with various combinations of muscle and skin from the adjacent abdominal wall (9–11) or from distant sites (12–18) have been described. Today, the most popular and useful muscles for the repair of abdominal wall defects are tensor fasciae latae (6, 12–14), sartorius (15), and rectus femoris (16, 17). These well-established flaps frequently cannot hold with the functional requirements of the abdominal wall because of their limitation in size, arc of rotation, frequent blood supply insufficiency and flap necrosis at the distal part of the flap, high donor site morbidity, and lack of controlled abdominal wall movement. However, it has been described that dynamic activity of the abdominal wall may be restored by using free microvascular innervated muscle flap. In 1998 Ninkovic at al. showed that a free functional (innervated) latissimus dorsi muscle (fLDM) was able to provide voluntary contractile activities in abdominal wall reconstruction (19). The purpose of this paper is to present our further surgical development for the reconstruction of large full-thickness abdominal wall defects using fLDM flap and to define the indication, advantages, and disadvantages of this technique, emphasizing the possibility of total restoration of physiological function.

## Materials and Methods

### Surgical Technique

**Figure 1:**
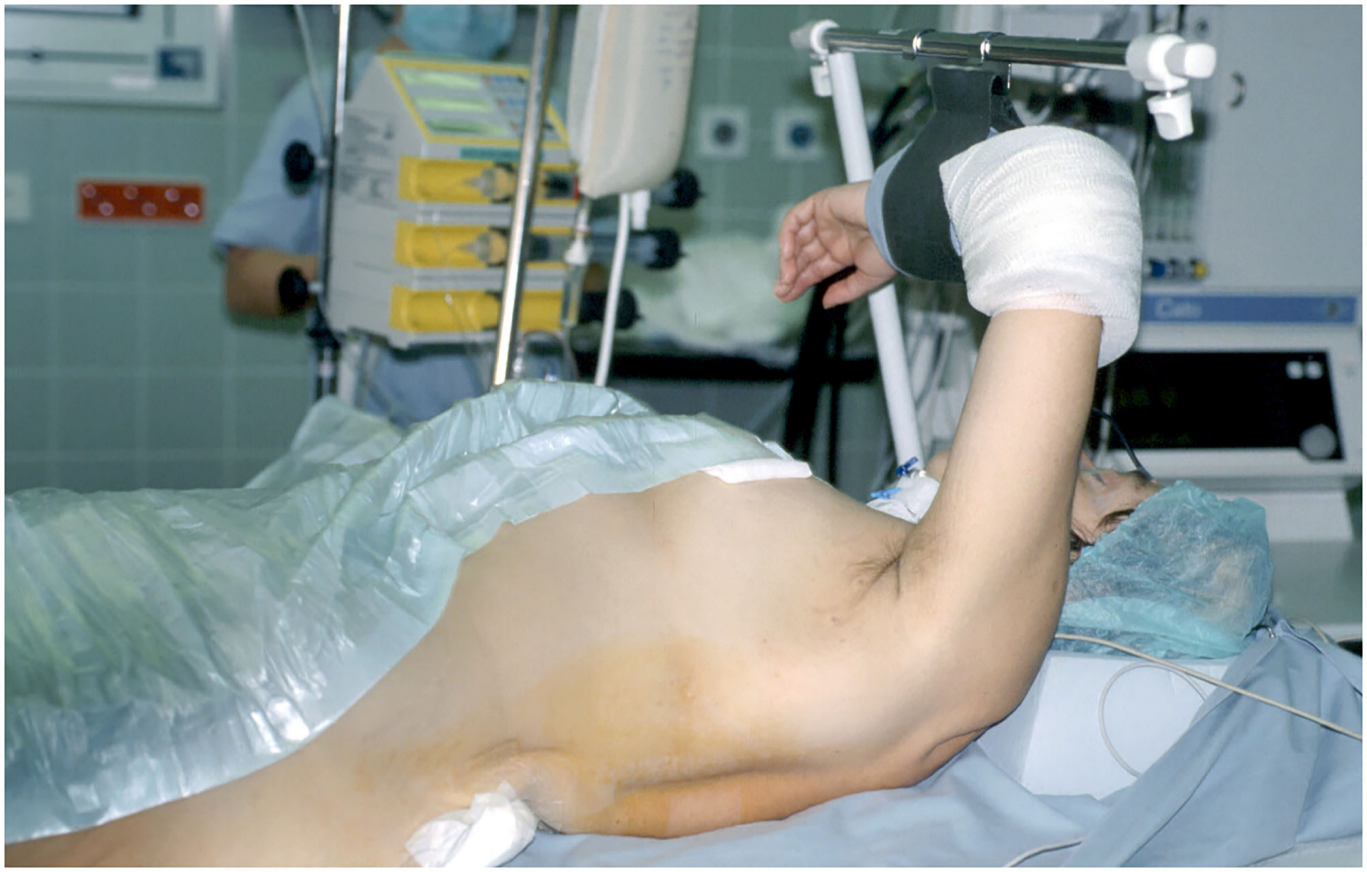
Special positioning of the patient.

**Figure 2:**
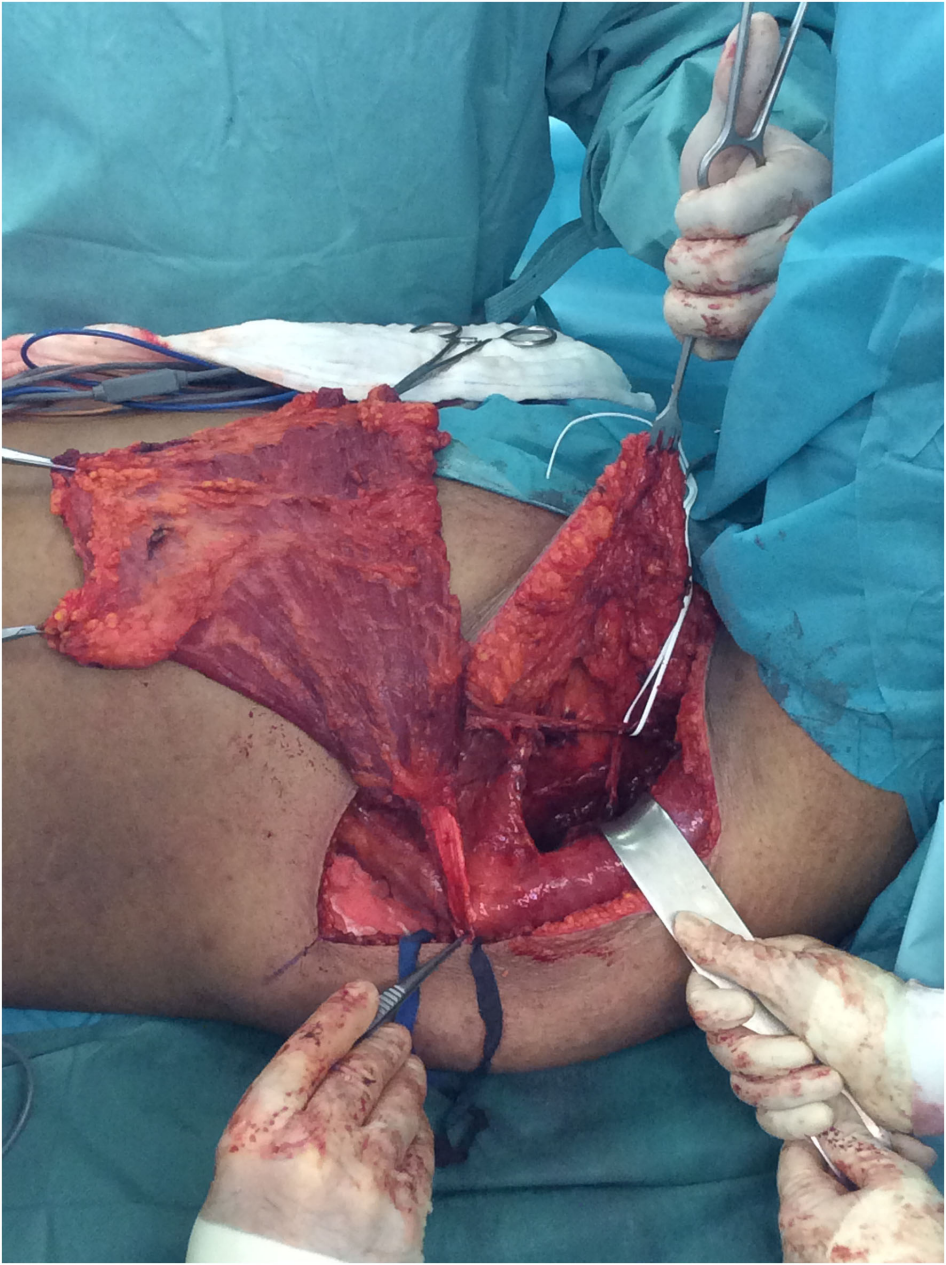
Complete elevation of the LDM except for the neurovascular bundle.

**Figure 3:**
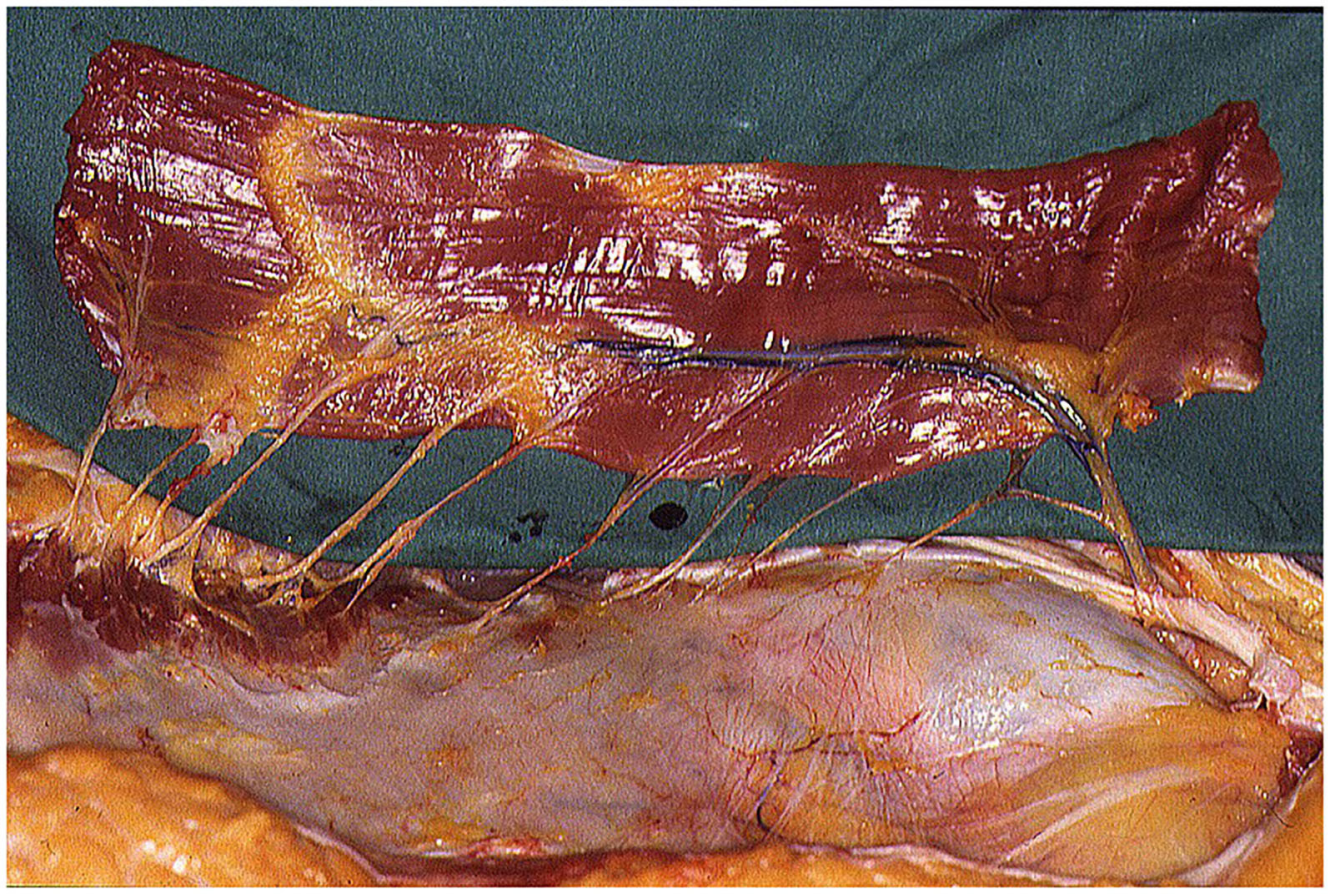
Fresh cadaver dissection shows innervation and blood supply of rectus abdominis muscle.

**Figure 4:**
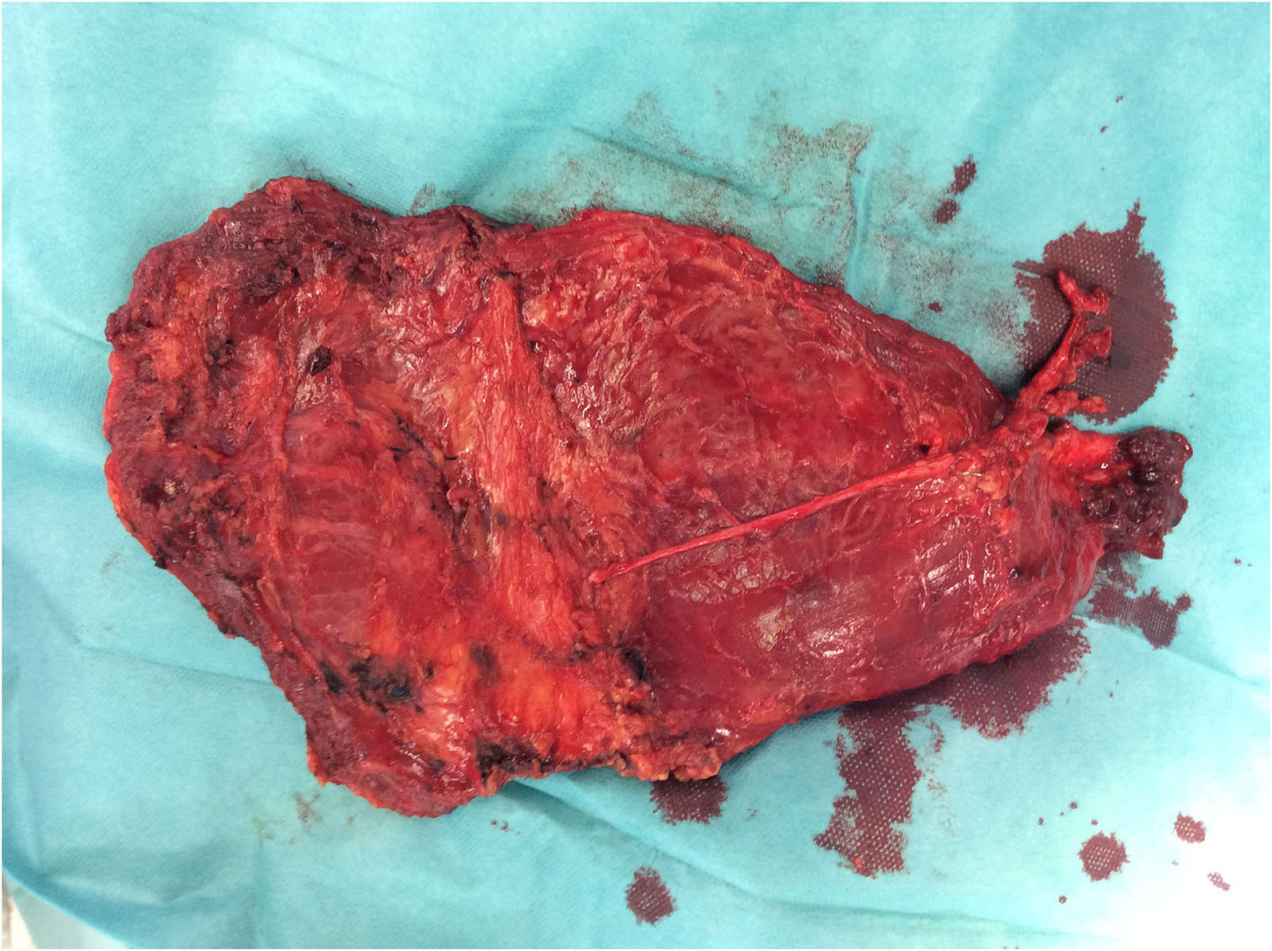
LDM free flap, with long thoracodorsal nerve, prepared for transfer in abdominal region.

**Figure 5:**
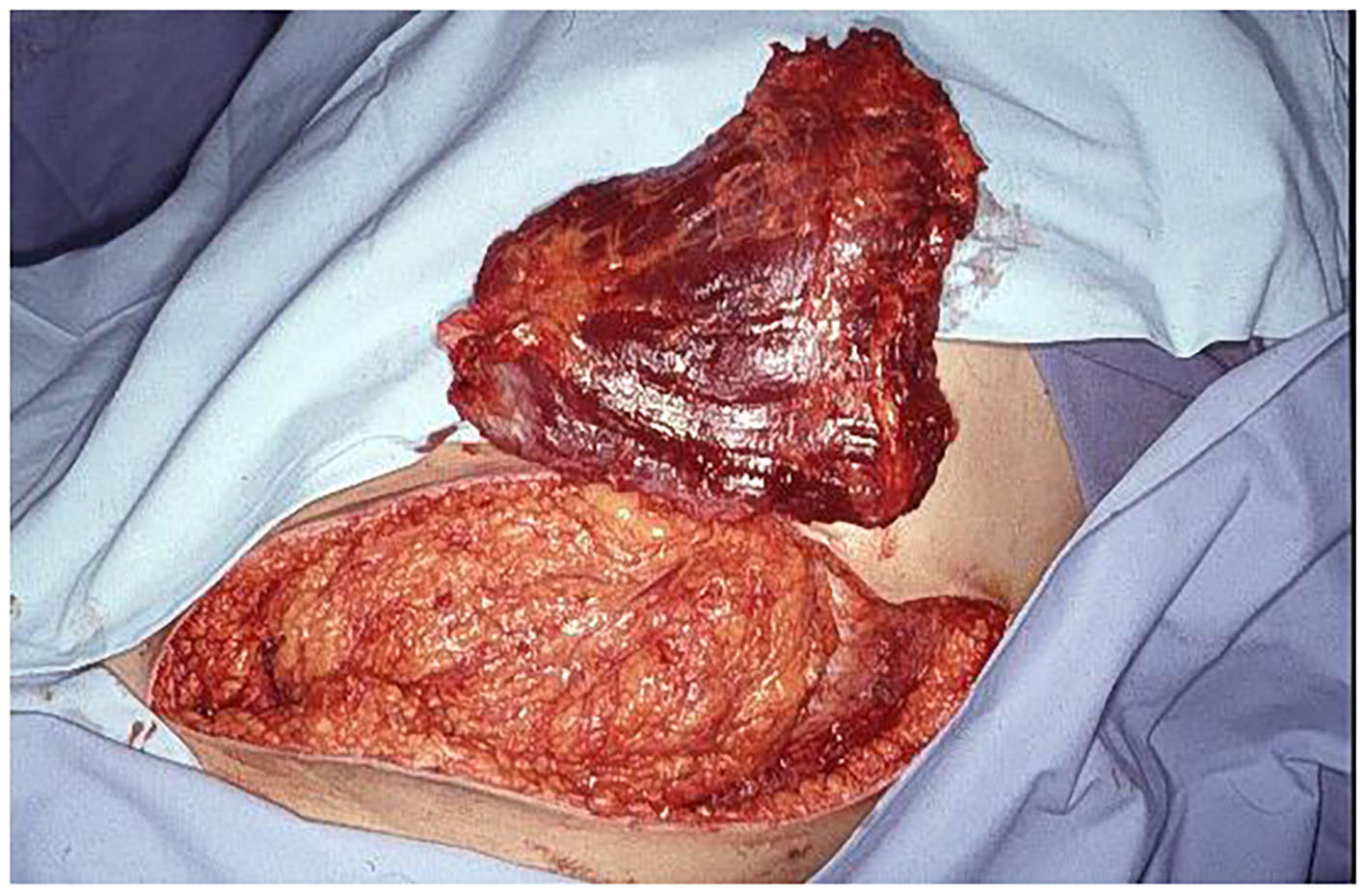
Free LDM is prepared for revascularization by inferior epigastric vessels and muscle insertion in abdominal wall defect.

**Figure 6:**
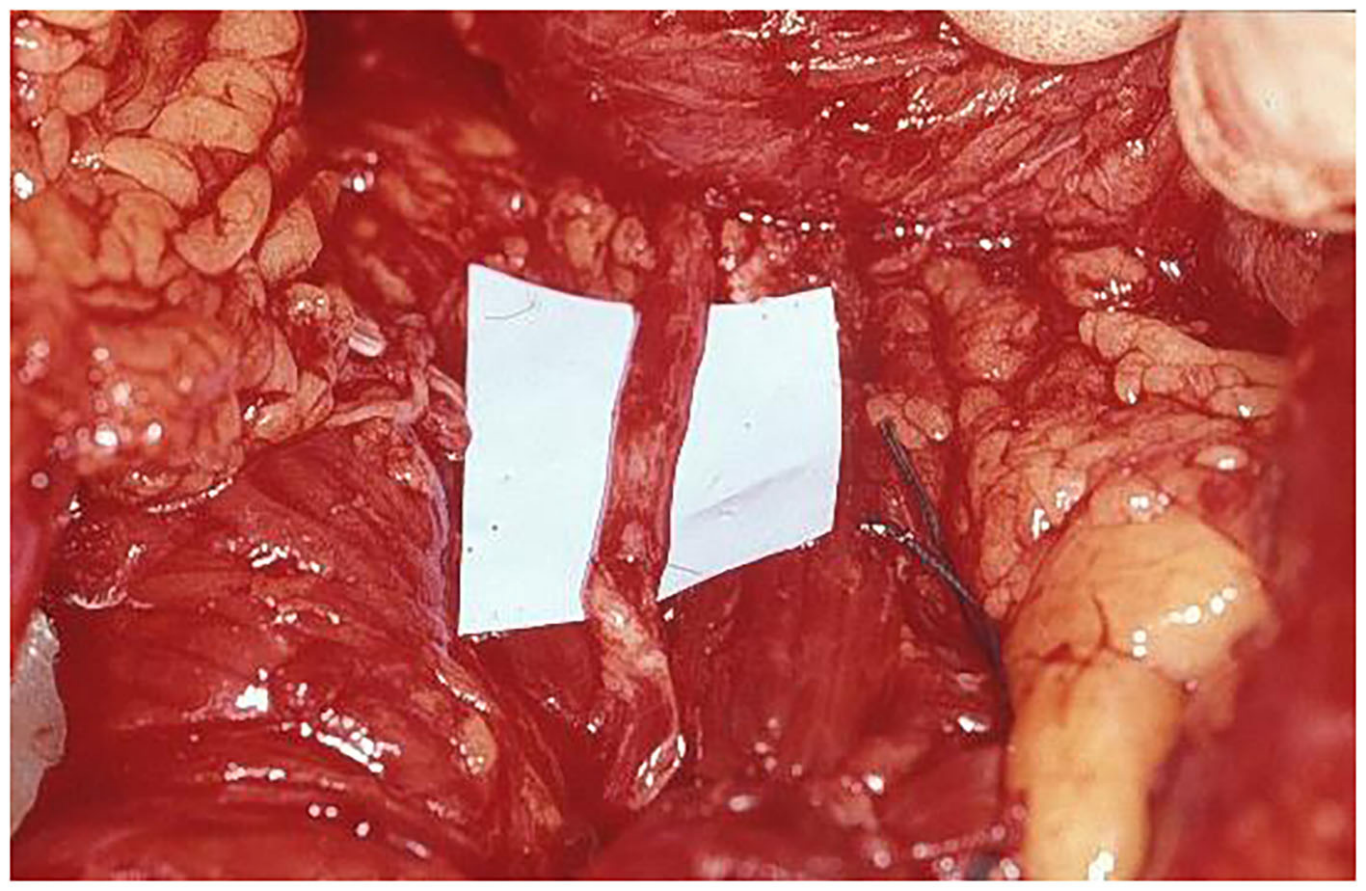
Nerve coaptation, with excellent nerve matching, between thoracodorsal nerve and motor branch of intercostal nerve supplying rectus abdominis muscle.

A simultaneous two-team approach was utilized in all patients to minimize operative time. Special positioning with the patient supine, the shoulder from which the muscle was harvested elevated, and the arm angled overhead was developed for the application of LDM for different indications (Figure 1). The latissimus dorsi muscle (LDM) of the nondominant arm was harvested by an axillary Z- incision. According to the reconstructive requirements, myocutaneous LDM or just muscle flap was harvested. During dissection of the LDM care had to be taken to mark the resting muscle tension by placing two sutures at defined muscle length *in situ* between the origin and insertion, prior to completing the dissection. This length of LDM muscle had to be restored at the abdominal recipient region during insetting of the myocutaneous flap to achieve full functional capacity of LDM. Any LDM retraction or shortening during insertion would reduce the contractile strength of the transplanted muscle. The LDM was completely elevated, except for the neurovascular bundle, which was not divided until the recipient vessels and nerve had been prepared for microanastomosis (Figure 2). At the same time, the second team prepared the abdominal wall for flap insertion. After identifying and severing the ipsilateral lowermost motor branches of the intercostal nerve (Figure 3) as well as the inferior epigastric artery and vein supplying the rectus abdominis muscle, the thoracodorsal vessels and nerve were divided (Figure 4). The LDM was transferred to the abdomen (Figure 5), and a microsurgical vascular anastomosis was performed immediately. Inferior epigastric artery and vein were used in 12 from the 14 cases. In one case the abdominal wall defect was more cranial, therefore the vascular vessels could be anastomosed to the superior epigastric artery and vein, but this can be a challenging procedure because of the diameter of the recipient vessels. Alternatively, the internal mammary vessels with vascular graft can be reached, which was performed in one case. The muscle ischemia time should not exceed 3 h as in this procedure the revascularization is usually performed within 40 min. In the abdomen the transferred free LDM was attached to the rigid surrounding fascial structures, additionally to the scarring muscles and linea alba. The original resting tension of the LDM was restored by longitudinal tension of the muscle fibers, rebuilding normal muscle length measured at the donor site. In the final step, microsurgical coaptation of the thoracodorsal nerve to the previously identified lowermost branches of the intercostal nerve was performed under the microscope using 10/0 monofilament nonabsorbable sutures (Figure 6). We exclusively used the motor part, selected by nerve stimulation, of the intercostal nerve, supplying the rectus abdominis muscle, mainly at the umbilicus level to have good input and matching with the thoracodorsal nerve. In four cases we used two intercostal nerves to increase motor nerve growth. In the first postoperative week, perfusion of the transferred LDM was monitored with an intramuscular probe measuring the tissue pO2 (Licox^®^).

### Patients

**Figure 7:**
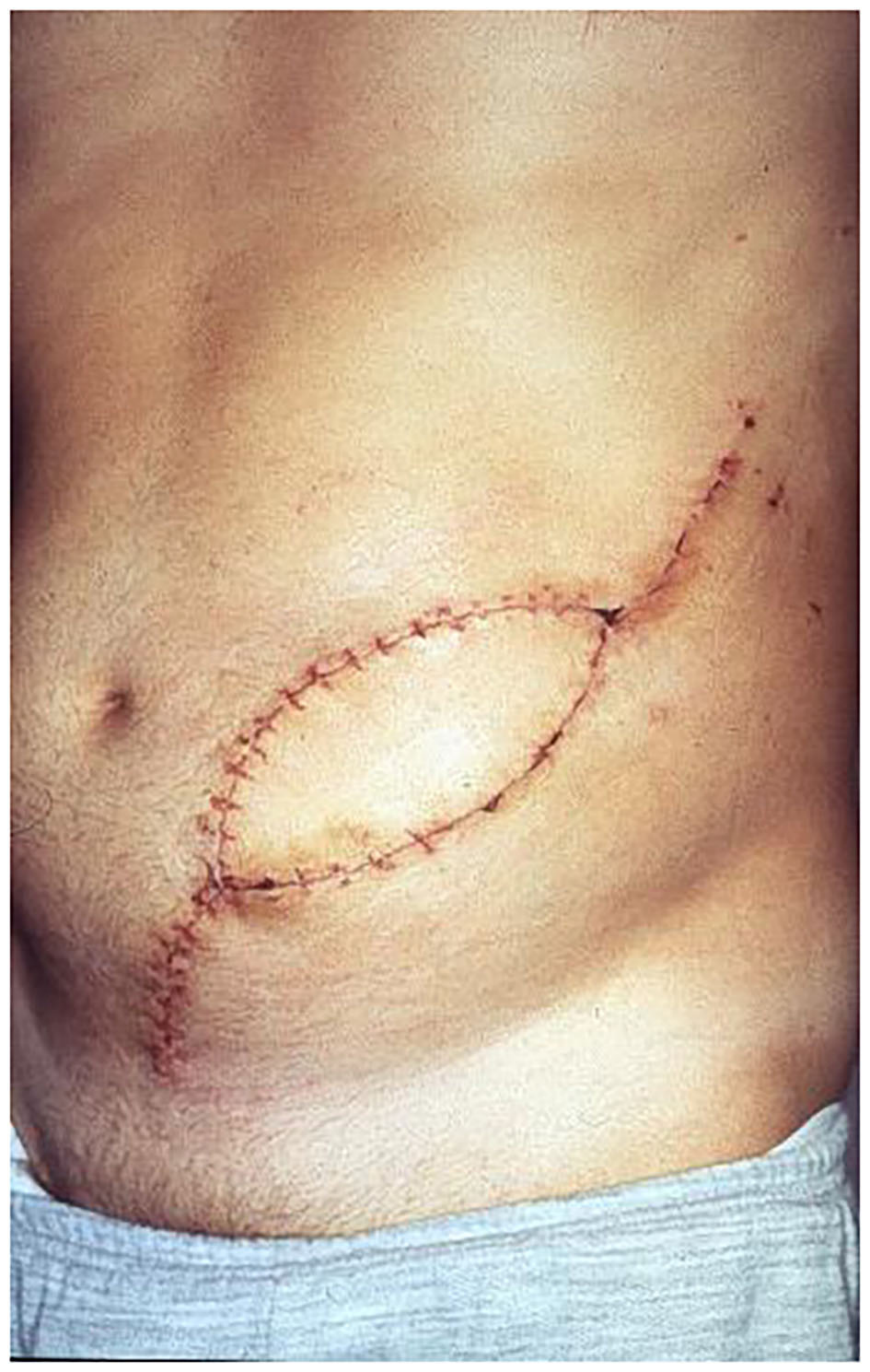
Patient 2 weeks after surgery and stitch removal with fLDM.

**Figure 8:**
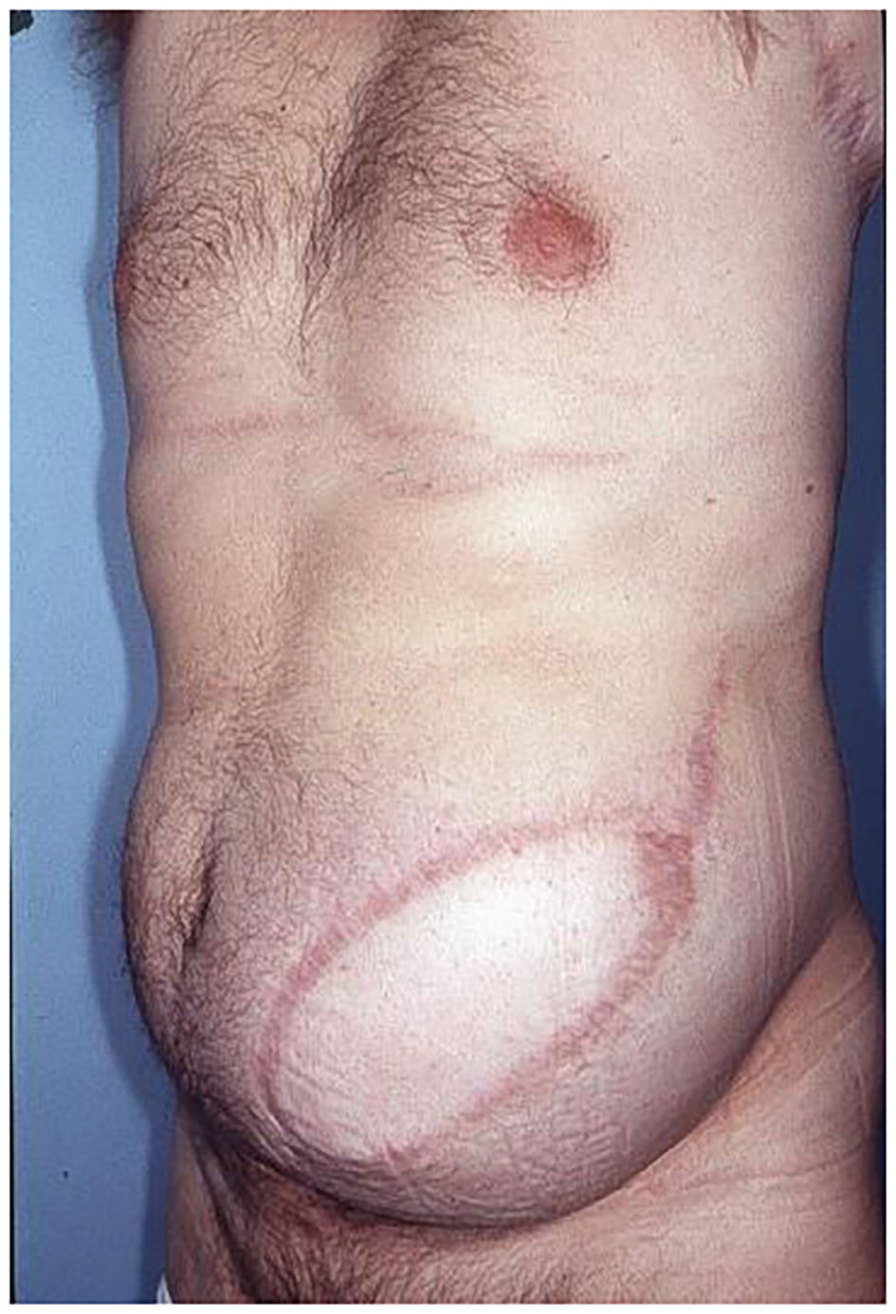
Patient 2 years after abdominal wall reconstruction with fLDM.

Between August 1992 and September 2019 fourteen patients with large full-thickness abdominal wall defects underwent abdominal wall reconstruction using a fLDM flap. The patients' age at the time of surgery ranged from 11 to 72 years (mean 41 years). Most patients needed reconstruction after primary trauma (6 patients, 43%). In four cases, abdominal wall reconstruction was performed immediately after tumor resection. In the first case in 1992, abdominal wall was reconstructed just by an fLDM flap without any synthetic mesh support. The reconstructed abdominal wall shows permanent weakness at the site of LDM. Therefore, three cases were reconstructed simultaneously with tumor resection using synthetic mesh for abdominal wall stabilization and a free fLDM flap for coverage and functional dynamic reconstruction (Figures 2, 4–8). However, in the fourth case, primary sarcoma resection was performed by general surgeons and in a second operation a week later, when the patient was presented to the plastic surgeon for the first time, abdominal wall reconstruction was achieved by fascial graft and a fLDM flap. In eight cases a two-stage procedure was performed. Primary defect coverage was obtained with a split-thickness skin graft (STSG). A definitive reconstruction was achieved by dermabrasion of the skin graft. The residual dermal layer supplied sufficient abdominal wall strength, therefore there was no need to use a synthetic or a biological mesh. The coverage was followed with a fLDM flap. This combination provides a strong vascularized fascia-like repair as well as a well-vascularized overlying soft-tissue coverage (19). All patients' details are shown in Table 1.

**Table 1:** Patients' characteristics.

**No**.	**Sex (%)**	**Age**	**Etiology (%)**	**Size of defect (cm)**	**Reconstruction (%)**	**Complications (%)**
1.	M	38	DFSP	24 × 16	One stage fLDM	Abdominal wall weakness
2.	M	17	Trauma	28 × 25	Two stage STSG+ fLDM	None
3.	F	51	Rhabdomyosarcoma	25 × 14	Two stage STSG+ fLDM	None
4.	F	63	High-grade myxoid Sarcoma	20 × 18	One stage Synthetic mesh + fLDM	Died 2 months after Reconstruction
5.	M	11	Small bowel transplantation	24 × 22	One stage fLDM	Wound dehiscence donor site
6.	M	32	Trauma	34 × 18	Two stage STSG+ fLDM	Minor
7.	F	41	DFSP	16 × 11	Two stage STSG+ fLDM	None
8.	M	45	Trauma	25 × 14	Two stage STSG+ fLDM	Minor
9.	M	29	Trauma	18 × 12	Two stage STSG+ fLDM	None
10.	F	30	Rhabdomyosarcoma	22 × 14	Two stage STSG+ fLDM	None
11.	M	72	Pleomorphic liposarcoma	26 × 16	One stage fascial graft+ fLDM	Minor
12.	F	32	DFSP	16 × 10	One stage synthetic mesh + fLDM	Minor
13.	M	56	Trauma	17 × 12	One stage synthetic mesh + fLDM	None
14.	M	52	Trauma	22 × 14	Two stage STSG+ fLDM	Minor
all	F=5 (36)	mean = 40.64 SD = 17 range = 11-72	DFSP = 3 (21) Trauma = 6 (43) Rhabdomyosarcoma = 2 (14) Myxoid sarcoma = 1 (7) Small bowel transplantation = 1 (7) Pleomorphic liposarcoma = 1 (7)	mean = 22.64 SD = 5 range = 16-34 × mean = 15.43 SD = 4 range = 10-25	Two stage STSG+ fLDM = 8 (57) One stage fLDM = 2 (14) One stage synthetic mesh + fLDM = 3 (21) One stage fascial graft+ fLDM = 1 (7)	None = 6 (43) Minor = 5 (36) Death 2 months after Reconstruction = 1 (7) Abdominal wall weakness =1 (7) Wound dehiscence donor site = 1 (7)

*DFSP, Dermatofibrosarcoma protuberans; STSG, Split thickness skin graft; fLDM, Functional Latissimus Dorsi Muscle; minor complications, donor site seromas, treated with punction and Doxycycline*.

#### Case

**Figure 9:**
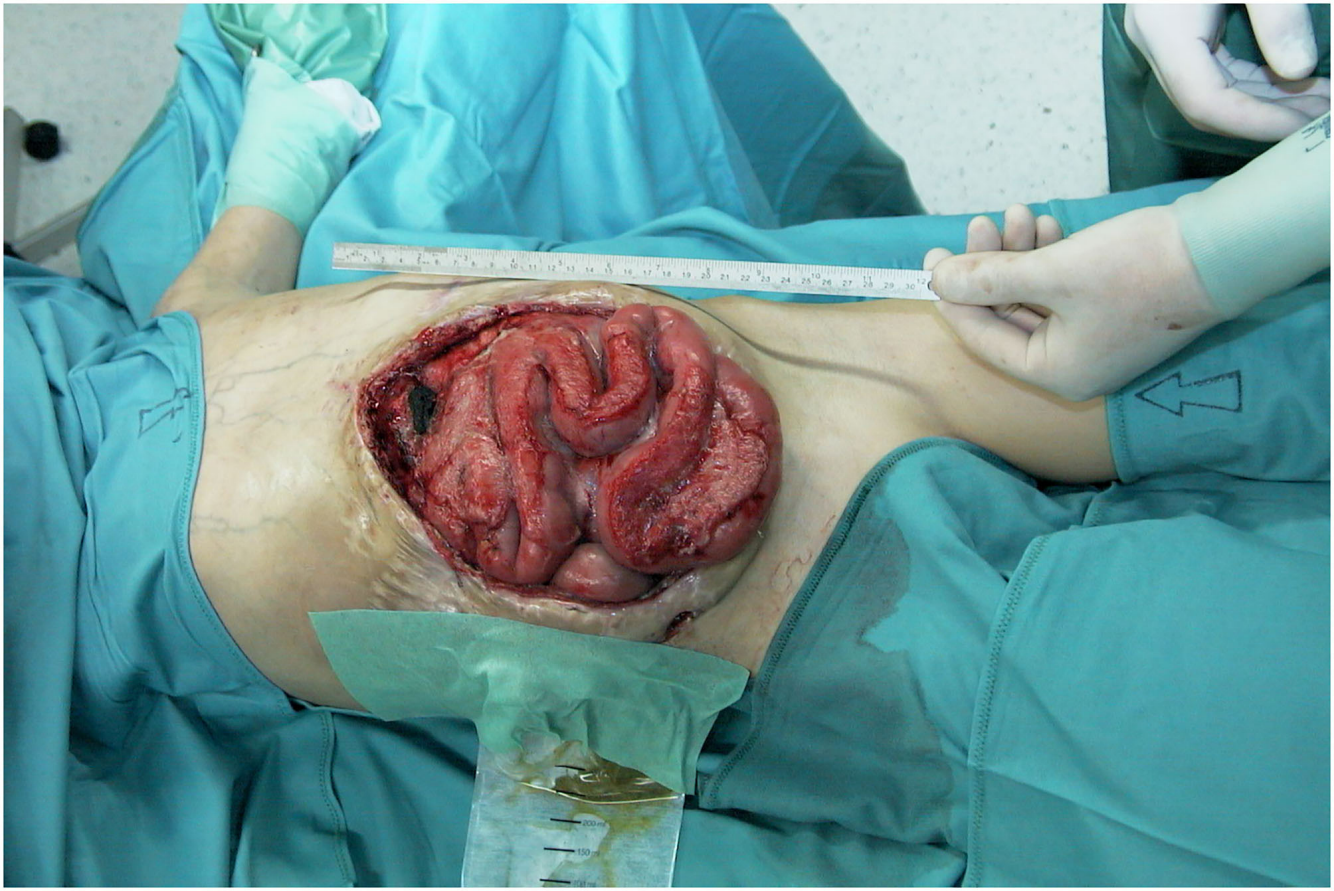
Preoperative view of the immense abdominal wall defect (24 cm × 20 cm) of an 11 years old boy after second bowel transplantation.

**Figure 10:**
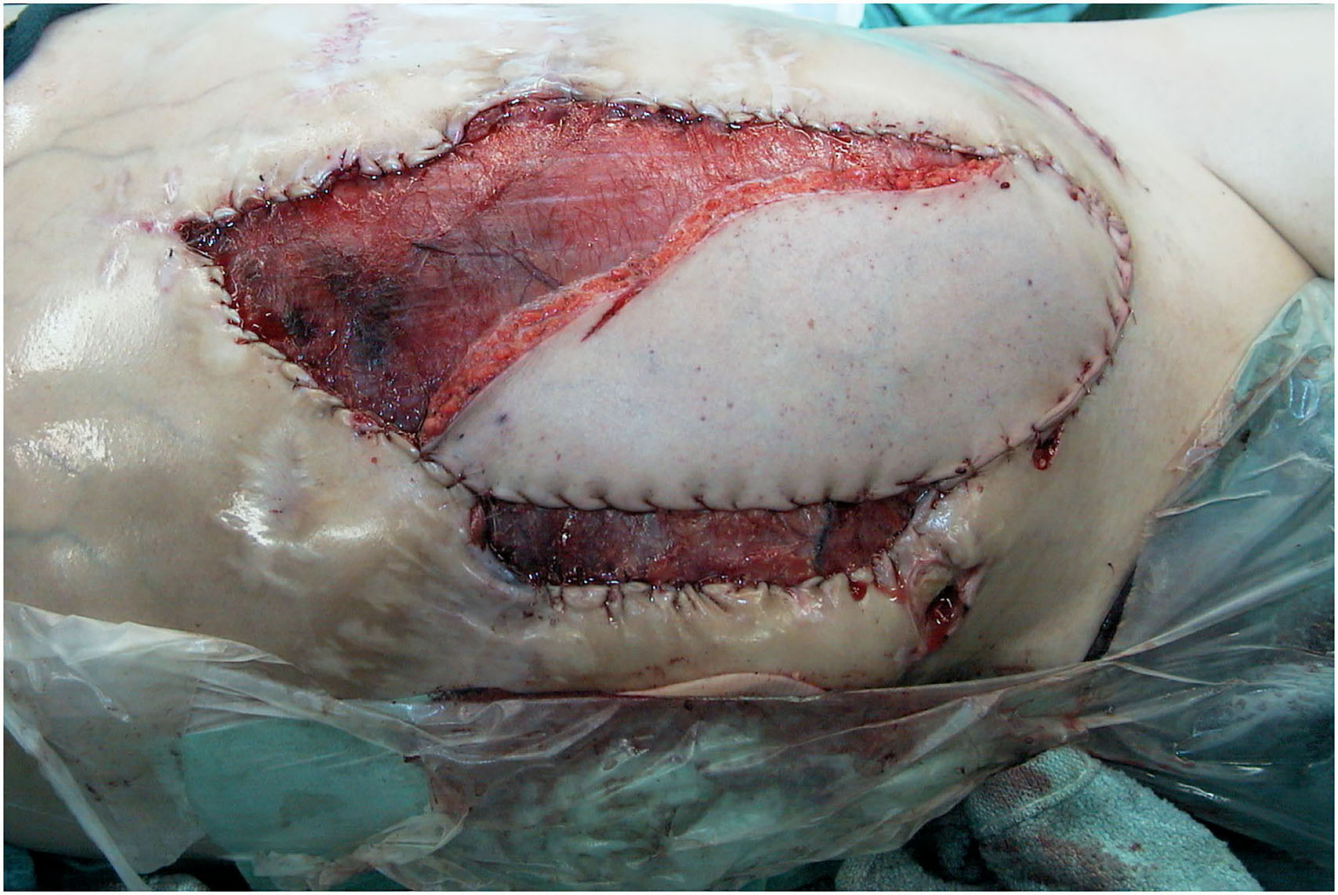
Intraoperative view at the end of insetting of the fLDM flap.

**Figure 11:**
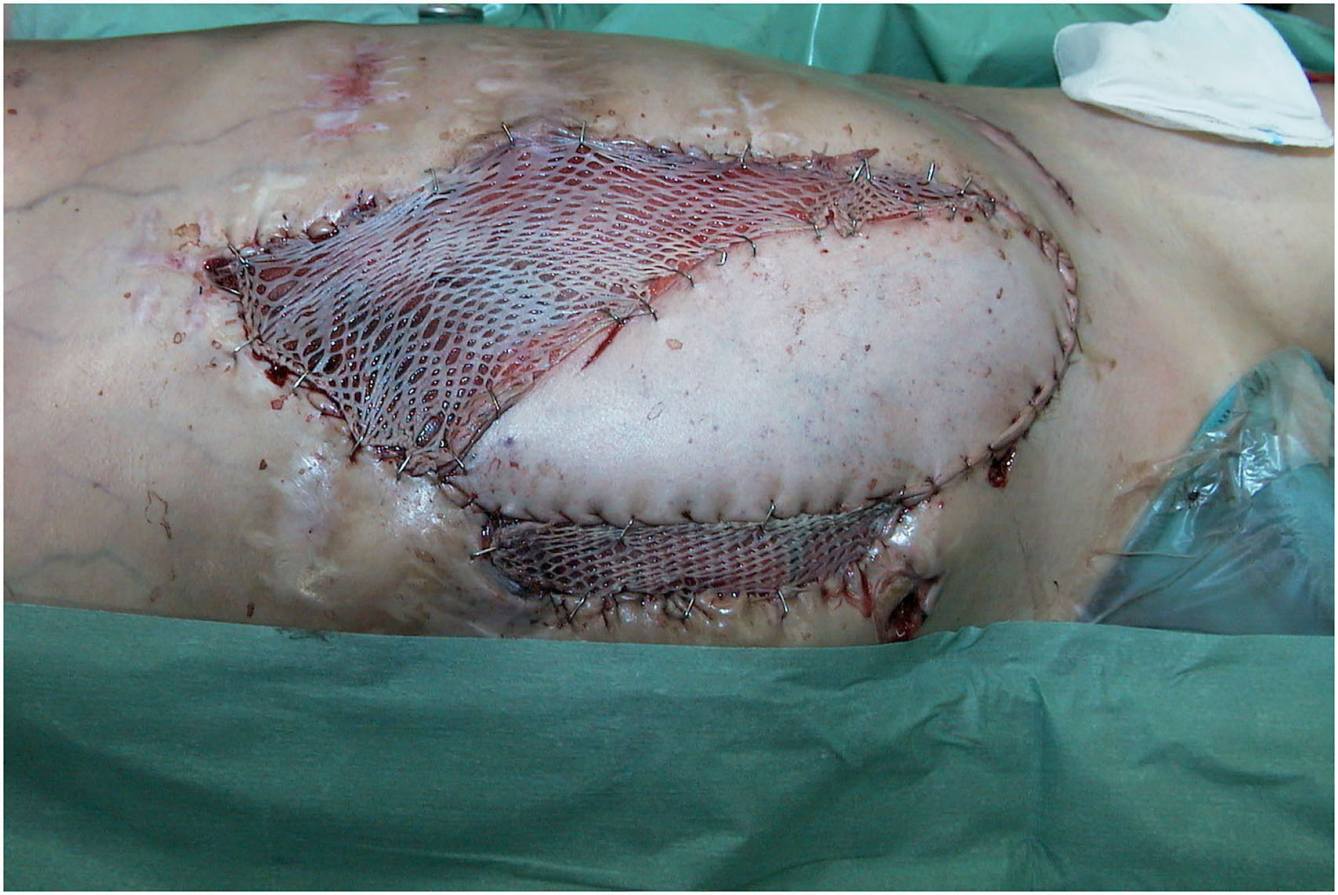
Intraoperative view after skin graft mesh coverage of the exposed LDM flap.

**Figure 12:**
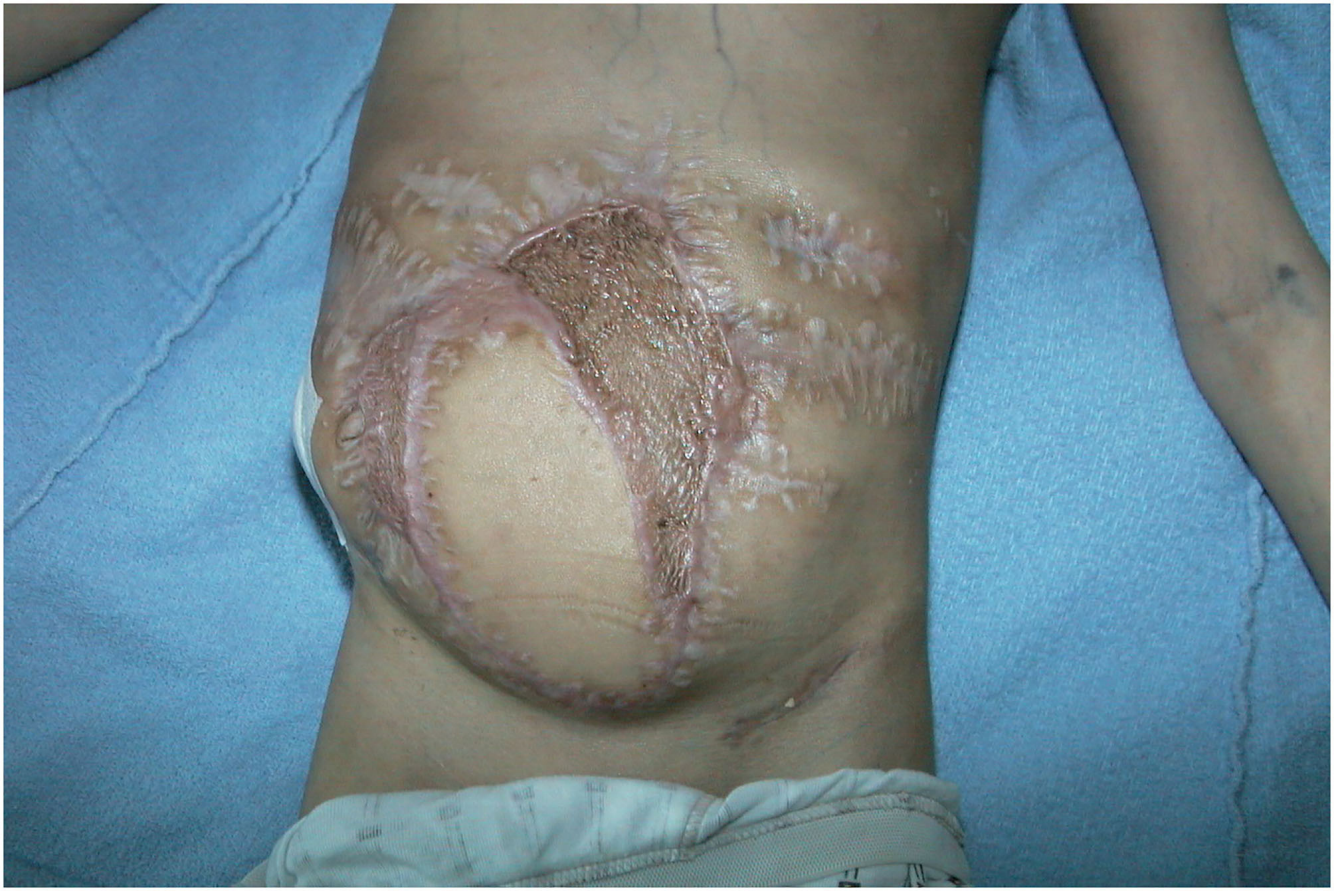
Three months follow-up after LDM transfer without any wound healings problem at recipient site.

**Figure 13:**
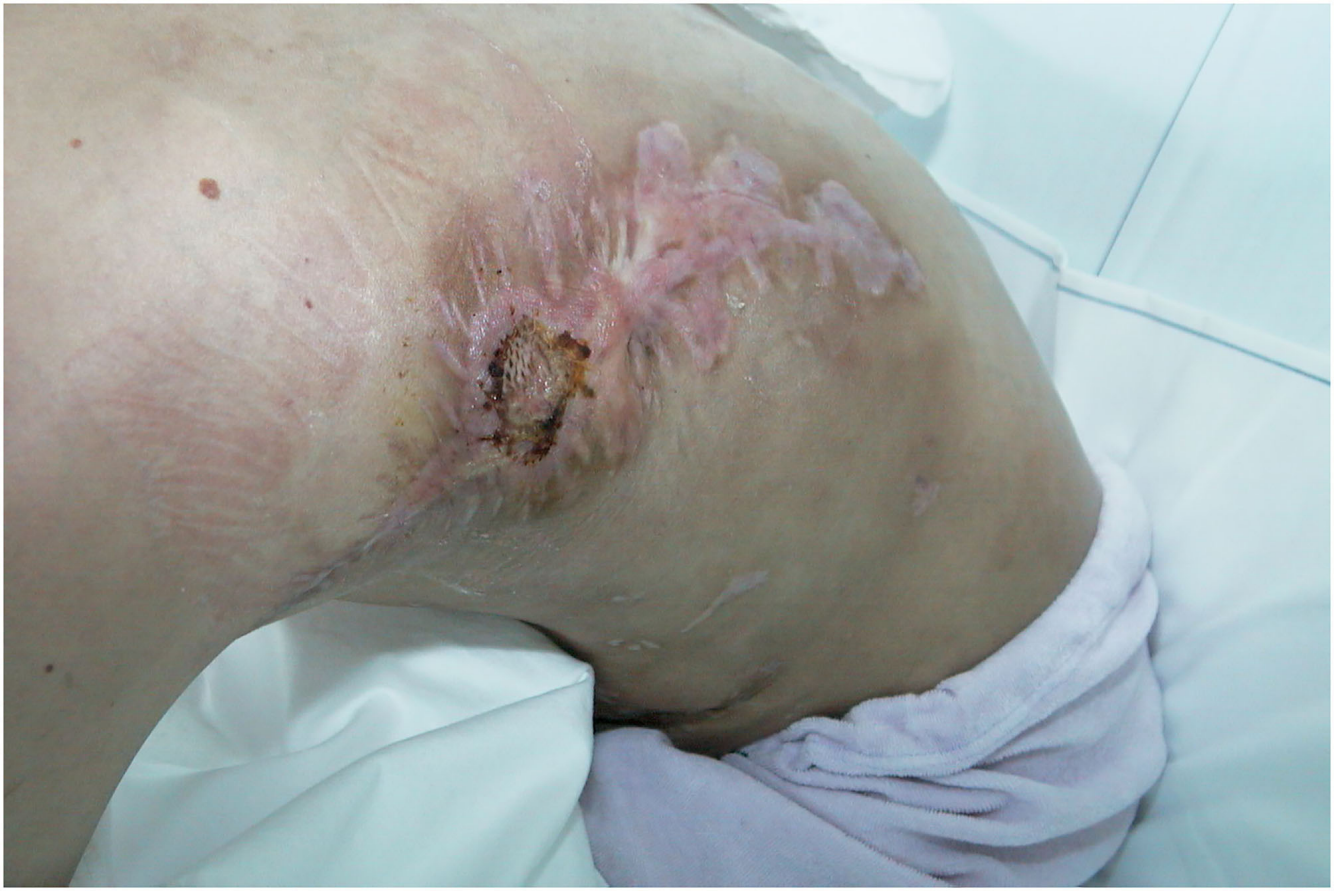
Minimal wound dehiscence at donor site on 3 months follow-up treated with split thickness skin graft (STSG).

In January 2000, an eleven-year-old boy underwent a small bowel transplantation. After 3 weeks of primary small bowel transplantation, a second transplantation was indicated. After the second small bowel transplantation, an immense abdominal wall defect caused by severe swelling, infection, and unfitting volume of the donor small bowel, provided by his mother, was developed (Figures 9–13). The young patient was in very bad general condition, under immunosuppressive therapy, with severe abdominal infection and high septic temperature at the time when he was presented to the plastic surgeons for the first time. To reduce the risk of infection and provide a functional reconstruction, we performed a radical soft tissue debridement and an abdominal wall reconstruction with a fLDM flap in this life-threatening situation. Revascularization of LDM flap was achieved by anastomosis to deep inferior epigastric artery and vein, and reinnervation was achieved by nerve coaptation between the motor branch of the intercostal nerve supplying rectus abdominis muscle and the thoracodorsal nerve. Due to the limited size of the skin island on the LDM (Figure 10), the exposed part of the LDM muscle was covered with mesh skin grafts taken from the upper leg (Figure 11). Synthetic or biological mesh was not used because of the severe infection. The boy had a mostly uneventful postoperative recovery. At the 3-month follow-up, the boy had a completely closed abdominal wall without any wound healing problems (Figure 12). He suffered from a small wound dehiscence at the donor site without any signs of infection (Figure 13). This dehiscence was treated with a STSG without further wound healing problems. The last follow-up examination was 6 months after abdominal wall coverage, showing stabile coverage without any wound healings problem.

### Postoperative Considerations

The free flap should be monitored, according to protocol, during the first seven postoperative days. Patients should wear a supportive abdominal compressive dressing, ideally, until full muscle reinnervation has occurred, which takes in between 6 and 12 months, depending on the distance of coaptation site and entrance of the nerve into LDM. The nerve generally regenerates 1–2 mm per day, depending mostly on the patients' age. Strenuous exercises and heavy lifting of more than 10–15 pounds should also be avoided during this time. Following the initial wound healing phase, the patients should undergo a rehabilitation program that includes muscle electrical stimulation, breathing exercises, core-strength exercises, scar control, and an EMG follow-up. Depending on the degree of muscle regeneration, muscle training is intensified to obtain the necessary strength of contraction. Regular electromyography (EMG) provides important information about muscular reinnervation and resumption of the function of the LDM.

## Results

**Figure 14:**
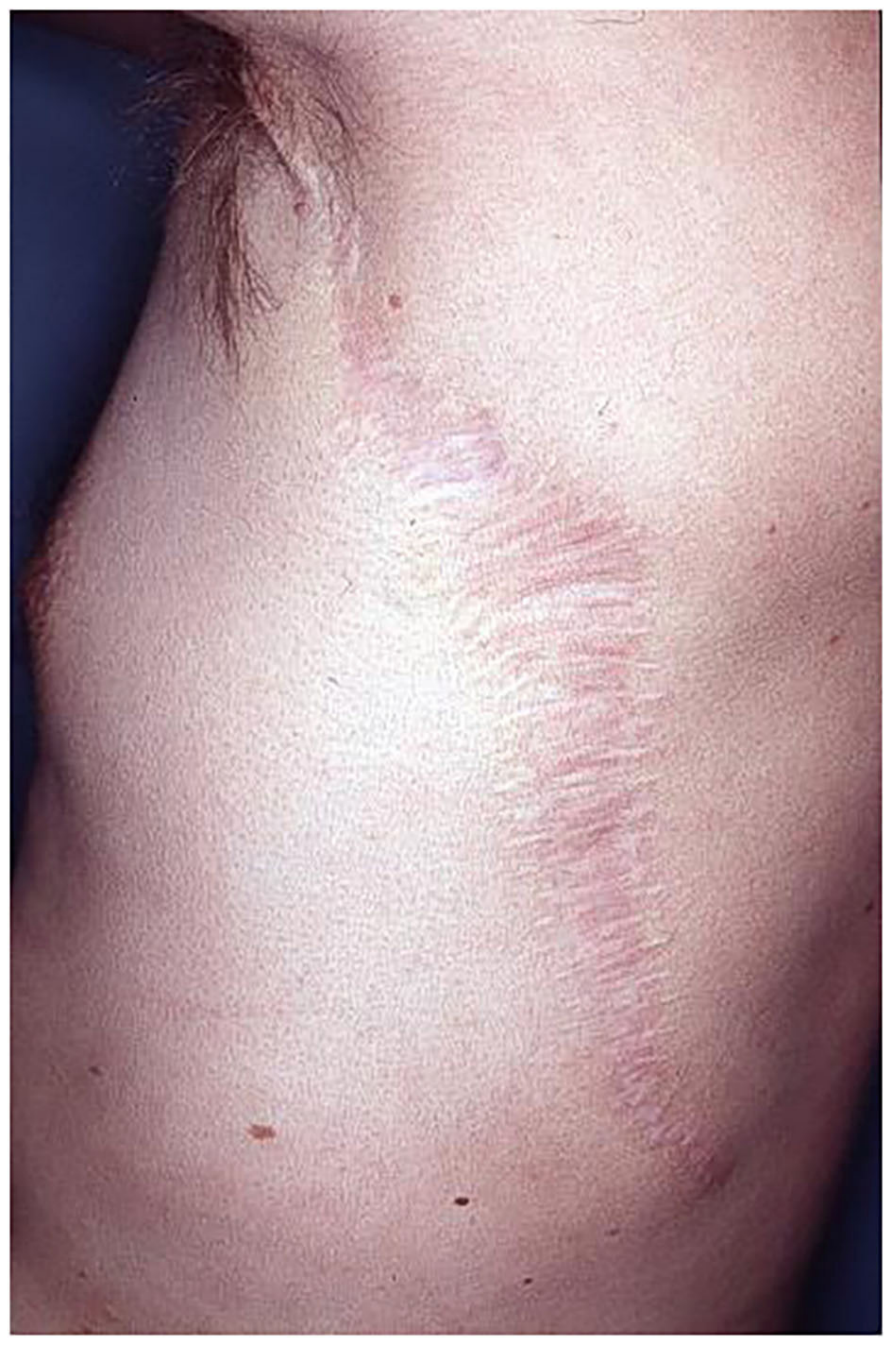
Donor site of the patient, 2 years after harvesting of LDM.

Altogether fourteen patients underwent full-thickness abdominal wall reconstruction using an fLDM flap. Twelve of the fourteen patients were alive at the last update, one patient died 2 months postoperatively due to sarcoma progression, and the young patient after small bowel transplantation died 14 months after LDM due to chronic organ rejection. The patients have currently been followed up for at least 26 months. There were no flap failures. One Clavien-Dindo 3b complication occurred. Due to a venous anastomosis thrombosis in the first 12 postoperative h, a flap revision was performed. No further major complications occurred. Minor complications were five donor site seromas that were treated with puncture and direct Doxycycline injection, and one wound healing problem at the donor site, which was treated with a STSG. The average hospital stay was 14 days (range 8–26 days). No abdominal wall weakness or hernias have occurred postoperatively, apart from the first operated patient who developed abdominal wall weakness after significant weight gain 6 months after surgery. Abdominal wall weakness was corrected by muscle tightening and additional support by synthetic mesh, which was not used in the primary reconstruction. Donor site scars and morbidity were accepted very well by all the patients. To determine reinnervation of transplanted LDM flap, EMG testing was performed at 3-month intervals in all patients postoperatively. In 12 of the 14 examined patients 1 year after abdominal wall reconstruction, EMG revealed complete reinnervation of LDM with voluntary muscle contraction. Following a special rehabilitation program, the muscle strength and capacity of contractility significantly increased in the first 18 months after reconstruction. Examination with a Cybex II device 18 months after reconstruction revealed synergistic function between the transferred LDM and the remaining abdominal wall muscles. Furthermore, it showed that there is enough strength of the transferred LDM to maintain abdominal wall stability through its own contractility. The patients' postoperative results are presented in Figures 7, 8, 11–13, 14.

## Discussion

The aim of the reconstruction of a complex full-thickness abdominal wall defect is the regain of functional stability, structural integrity, protection and retention of internal organs, as well as physiological muscle contraction. The reestablishment of anatomic structures and the synergistic innervations of the transplanted muscle and remaining abdominal muscles are the main principles for functional abdominal wall reconstruction. By using an innervated fLDM, we have gained well-defined goals. This dynamic abdominal wall reconstruction maintains physiological abdominal wall movement and normal chest wall extension during the breathing process. Therefore, there are no functional limitations of the reconstructed abdominal wall. By coaptation between the thoracodorsal nerve of LDM and the intercostal nerves, which supply the rectus abdominis muscle, the physiological reinnervation of the LDM is provided. After regeneration of the nerve and reinnervation of the LDM, controlled by EMG, the reinnervated LDM can take over the missing abdominal muscle function. Additionally, voluntary synergistic contractility of the abdominal wall muscles is reestablished, which represents a functionally and esthetically superior result, compared to any other muscle transferred from the upper leg (12, 14, 15, 17).

The etiology of abdominal wall defects can be divided into congenital and acquired ones (6). The acquired defects can be the result of large hernias, primary or secondary tumor resection, severe abdominal trauma, or abdominal wall infection. The timing, primary or staged reconstruction, and method of reconstruction must be chosen depending on the etiology of the defect. Congenital abdominal wall defects can be treated successfully with preexpanded musculocutaneous tissue (20), providing both adequate coverage and autogenously innervated contractility. Primary repair is possible in the reconstruction of large hernias and after tumor resection. To reconstruct defects after abdominal wall resection for malignancy, a supportive mesh should be included to restore the fascia's stability. Soft tissue coverage is then provided with an fLDM flap. Severe traumatic injuries and abdominal wall infections or sepsis need to undergo staged reconstruction following adequate debridement and proper treatment of intraabdominal pathology. To achieve a temporary primary closure split-thickness, skin grafting to the abdominal viscera is used. Subsequently, after complete stabilization of the systemic and local conditions, definitive reconstruction can be performed. At the time of definitive reconstruction, deep skin graft dermabrasion, until the dermis layer, give us a facial-like layer with adequate strength to stabilize the static abdominal wall (19). This is the best way to avoid the use of synthetic mesh and its attendant complications (21, 22). This dermal layer must be supported by well-vascularized tissue to achieve stabile coverage. Using fLDM, we were not only able to repair soft tissue defects but also reconstruct voluntary contractility and a dynamic regular functional abdominal wall.

Well-established methods to repair lower abdominal wall defects are pedicled muscle or musculocutaneous flaps, like tensor fasciae lata (12–14), rectus femoris (16), or sartorius (15) as well as the extended latissimus dorsi muscle (23) for localized upper abdominal wall defects. All loco-regional options (24) for reconstruction have their limitations in arc of rotation, size, and dimension of a flap, and not at least functional and esthetic disadvantages at the donor site. The main problem is that in general the well-perfused proximal part of the flap does not reach the defect due to the loss of tissue at the pivot-point area so that the defect is covered by poorly perfused distal parts of the flap. Additionally, the pedicled muscle flap has to be denervated to gain greater movement to reach the defect. Cutting the nerve causes complete muscle atrophy and fibrosis, which leads to adequate coverage without any contractility and voluntary movement. If it is possible to keep some innervation of the pedicle muscle flap, the functioning part of the muscle flap does not have synergistic contractility with the remaining abdominal wall muscle. It leads to independent contraction according to the origin of the flap and its own innervation.

Transfer of a free microvascular flap is a well-established method in reconstructive surgery (25, 26). It provides the tissue with a rich blood supply, which improves the healing process, its resistance to infections, and quality of reconstruction. The technique provides freedom in flap design for optimal contour in accordance with size and shape of the defect. For the functional reconstruction in the region of damaged or missing muscles, innervated free muscle can be applied. Since 1970 when the first successful experimental work on functional muscle transfer was reported (27), a huge variety of concepts have been developed to restore facial expression (28, 29), improve extremity extension or flexion (30, 31), augment cardiac compression (32), or to restore detrusor function (33) by transplanting free muscle flaps with neurovascular anastomosis. The ideal donor muscle must present a sufficient cross-section and mass for exerting force, suitable fibers length for excursion, and appropriate tissue architecture and arrangement. Considering this, the gracilize muscle and the LDM fit the best for adequate abdominal wall reconstruction due to their anatomic arrangement of the muscle fibers within a strap muscle configuration, as opposed to that of a pennate configuration. The LDM is stronger and has got more volume than the gracilis muscle. With its reliable and suitable anatomy, the latissimus dorsi muscle is the most qualified to meet these demands. However, in functional muscle transfer, some decisive points have to be taken into consideration to avoid disappointing results. The most critical part of the functional muscle transfer is nerve selection and repair (30, 31, 34). First of all, the motor component of the recipient intercostal nerve has to be detected precisely and meticulous nerve coaptation should be placed as close as possible to the neuromuscular junction to minimize the time of nerve regeneration and keep muscle denervation phase short. The intercostal nerves that supply the rectus abdominis muscle are easy to dissect and match the thoracodorsal nerve very well. After 18 months, regeneration of the nerve and reinnervation of the transplanted muscle are completed. Being intensively trained, the latissimus dorsi muscle offers enough contractile capacity and strength to replace the function of the missing abdominal wall muscles. This is the reason why we have chosen the fLDM for the reconstruction of full-thickness abdominal wall defects. It provides the advantages of free-tissue transfer and has ability to reestablish active abdominal wall contraction.

In conclusion, we could say that an fLDM flap is able to meet the requirements of anatomic and dynamic function in reconstruction of complex abdominal wall defects. In complicated, infected abdominal wall defects staged reconstruction with skin graft primary, and secondary dermabrasion of this skin grafted region gives a strong and vascularized fascia-like layer, which in combination with fLDM, obtains optimal function and esthetic results.

## Data Availability Statement

The original contributions presented in the study are included in the article/supplementary material, further inquiries can be directed to the corresponding author.

## Ethics Statement

Ethical review and approval was not required for the study on human participants in accordance with the local legislation and institutional requirements. Written informed consent from the participants' legal guardian/next of kin was not required to participate in this study in accordance with the national legislation and the institutional requirements. Written informed consent was not obtained from the individual(s), nor the minor(s)' legal guardian/next of kin, for the publication of any potentially identifiable images or data included in this article.

## Author Contributions

MilomirN and MarijanaN contributed to the concept, literature review, manuscript writing, and revision of manuscript. MarinaN contributed to the rehabilitation section, manuscript writing and critical review of the article. DÖ provided a critical review of the article. All authors contributed to the article and approved the submitted version.

## Conflict of Interest

The authors declare that the research was conducted in the absence of any commercial or financial relationships that could be construed as a potential conflict of interest.

## Publisher's Note

All claims expressed in this article are solely those of the authors and do not necessarily represent those of their affiliated organizations, or those of the publisher, the editors and the reviewers. Any product that may be evaluated in this article, or claim that may be made by its manufacturer, is not guaranteed or endorsed by the publisher.
